# Assessment of the Role of Fire Regime as a Determinant of Habitat Selection by Medium‐Sized Grazers in a West African Savanna

**DOI:** 10.1002/ece3.73688

**Published:** 2026-05-17

**Authors:** Omobayo Ghislain Zoffoun, Chabi A. M. S. Djagoun, Côme Agossa Linsoussi, Etotépé A. Sogbohossou

**Affiliations:** ^1^ Laboratory of Applied Ecology (LEA), Faculty of Agronomy Sciences University of Abomey‐Calavi (UAC) Abomey‐Calavi Benin; ^2^ GeoEnvironmemt and Biodiversity Conservation (GeoEBC) NGO Cotonou Benin; ^3^ Senghor University Alexandria Egypt

**Keywords:** burned habitat, fire management, fire regime, soudanian savanna, ungulate

## Abstract

Fire is an important driver of habitat selection by herbivore species and savanna ecology. Several herbivores are threatened in West Africa and it's important to study how changes in habitat affect their selection behavior. The present study aims at assessing the fire role in determining the habitat selection by grazer species. We used total aerial counts of great mammals in the Pendjari Biosphere Reserve and extracted five grazer species data (hartebeest, kob, waterbuck, korrigum, and roan). We used negative binomial distribution to model grazer species' abundance in relation to fire, location, and NDVI. Our results reveal that the majority of grazer species studied displayed a strong occurrence inclination towards recently burned areas during the season, while waterbuck exhibited a different pattern, favoring areas in proximity to water sources, which are mainly unburned. Our analysis highlighted that the korrigum was found only in the core area, the Pendjari National Park (PNP), and the abundance model of kob and waterbuck was robustly impacted by the location in the reserve, with high abundance in the PNP. Additionally, the NDVI emerged as a non‐fire regime variable that significantly influenced grazer species abundance. Among fire regime variables, fire frequency emerged as a key variable impacting all species abundance, underlining its significance in shaping their abundance patterns. All grazer species studied showed positive abundance for recently burned areas, frequently burned, except kob, which showed aggregation around water sources. Our study provides valuable insights into how grazer species respond to fire regimes and habitat characteristics, shedding light on the complex interplay between these factors. These results can be used to better design a fire management plan in West African savannas which integrate habitat requirements for herbivores.

## Graphical abstract

Fire is an important driver of habitat selection by herbivores species and savannah ecology. The present study aims at assessing the fire role in determining the habitat selection by grazer species. Our results reveal that the majority of grazer species studied displayed a strong occurrences inclination towards recently burned areas during the season, while waterbuck exhibited a different pattern, favoring areas in proximity to water sources which are mainly unburned.

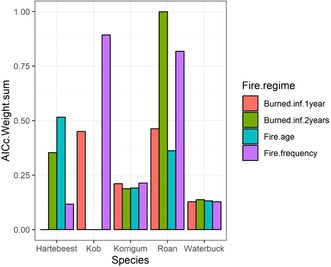

## 1 Introduction

Fire plays a crucial role in the ecology and evolutionary dynamics of grassland ecosystems (Fuhlendorf and Engle 2004). Fire is an important factor in grassland ecosystems, by stimulation of plant nutrients providing benefits to grazing mammals and variability in landscape features (Reich et al. 2001; Eby et al. 2014). Recent work across African savannas confirms that fire–grazer feedbacks are powerful at landscape scales, with large herbivores altering fuels and, in turn, fire regimes that structure subsequent habitat availability and selection (Karp et al. 2024). At finer scales, post‐fire regrowth strongly influences herbivores' space use and movement patterns in relation to the time elapsed since fire, with several species concentrating in very recently burned patches where forage quality peaks (Nieman et al. 2021; Masudi et al. 2024). On the other hand, fire reduces vegetation height and increases visibility, thereby potentially reducing predation risk to the herbivores (Eby et al. 2014). However, human pressures can modulate these responses: in multi‐system analyses, poaching curtailed herbivore ability to select optimal post‐fire forage, weakening classic pyric‐herbivory patterns (Brooke et al. 2020). Consequently, fire does not only directly influence ecosystem structuring, it potentially has a long‐term influence through the way ungulate species use their habitat (Fortin et al. 2005). The prevalence of fire in grassland ecosystems has resulted in the evolution of fire‐tolerant communities of plants and animals that depend on periodic burning for their existence (Olindo 1971), and fire is often used as a tool for the management of grassland vegetation (Edwards 1984). Beyond Africa, recent studies in Neotropical savannas and other fire‐prone savannas likewise show species‐specific habitat selection in relation to pyrodiversity and the proportion of recently burned versus long‐unburned areas, underscoring the generality of fire as a determinant of space use (Souza et al. 2023).

While developing conservation strategies for wildlife species it's essential to identify the preference and quality of different habitat types (Illius and Gordon 1992). Particularly, herbivore species consider the availability of habitat that provide a maximum forage intake (Schuette et al. 2016). Many factors can determine the spatio‐temporal distribution of herbivores species in savanna ecosystems. Among those factors, resources availability (Bell 1971; Mobæk et al. 2009; Ogutu et al. 2014), predation risk (Hopcraft et al. 2010; Vanderlocht et al. 2025), fire (Sankaran et al. 2008; Hassan and Rija 2011; Eby et al. 2014), vegetation height and cover (Massé and Côté 2009; Bjørneraas et al. 2012), human presences, hunting and livestock density (Vavra 2005; Sitters et al. 2009). Adding to this evidence base, continent‐wide and site‐level analyses, recent studies show that variation in fire frequency, fire age, and recent burns consistently predicts where and when grazers concentrate, with clear implications for habitat selection and management planning (Brooke et al. 2020; Nieman et al. 2021; Souza et al. 2023; Karp et al. 2024; Masudi et al. 2024). Large ungulate species commonly experience marked seasonal fluctuations in body condition driven by temporal variation in the availability of key resources such as water and forage (Fuentes‐Allende et al. 2023). During the dry season, reduced forage quantity and quality often result in weight loss and depletion of fat reserves. These seasonal patterns of nutritional stress are therefore expected to influence habitat selection and space use by grazers, particularly in relation to recently burned patches that provide high‐quality forage.

However, while the responses of different herbivore species to burning have been studied extensively in East and southern African savannas (Vermeire et al. 2004; Sensenig et al. 2010; Allred et al. 2011; Eby et al. 2014), less attention has been paid to West African assemblages despite the rich diversity of ungulates and environmental (climate, vegetation) uniqueness of this region (Kassa et al. 2007; Assédé et al. 2012). The role of fire in structuring these assemblages, essential for a good management of these rangelands, is even less well known. The vegetation of the Pendjari Biosphere Reserve, which hosts among the highest density of many large mammals in West Africa including buffalo, elephant, and lion, is strongly marked by the use of fire. The reserve also hosts endangered ungulate species according to the IUCN red list, the korrigum (*Damaliscus lunatus korrigum*) as well as hartebeest (*Alcelaphus bucelaphus major*), impacted by annual fire on the reserve. Although how the grazer species (mainly impacted by fire effects) respond to different fire regimes in the ecosystem is not well known. Most of the species are impacted by the secondary effects of burning on the ecosystem and are far more important to animals than the fire itself, especially changes in habitat as vegetation recovers from a fire (Masudi et al. 2024). Consequently, fire regimes can be key factors of great importance in the habitat use and distribution of ungulate species. In that context, resolving patterns of habitat selection at post‐fire regrowth within West African herbivore assemblages, especially grazer species, will contribute to our understanding of grazer species communities in general and help to recommend a better fire management plan in Pendjari Biosphere Reserve.

In this research, we delved into the influence of fire on the habitat utilization patterns of grazer species within the Pendjari Biosphere Reserve, situated in the Sudanian savanna. Our primary objectives included (i) identifying the frequency of grazer species occurrences in areas that had experienced recent burns, (ii) determining the most influential fire regime factors and non‐fire factors affecting grazer species, and (iii) assessing the relationship between fire regime variables and grazer species abundance. We hypothesized that recently burned areas would exert a positive impact on grazer occurrences and abundance due to potentially enhanced foraging opportunities (better quality), reported by Nieman et al. (2021).

## 2 Materials and Methods

### 2.1 Study Site

**FIGURE 1:**
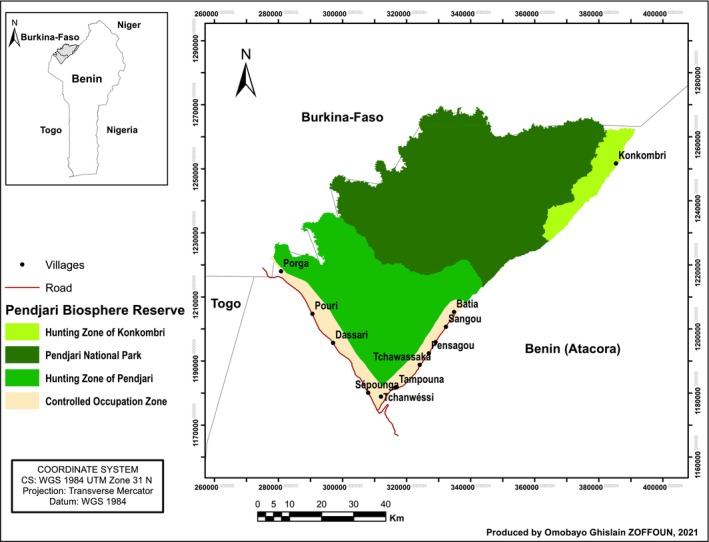
Study area, Pendjari Biosphere Reserve location in Benin.

The research was conducted within the Pendjari Biosphere Reserve (PBR), situated in north‐western Benin (coordinates 10°30′–11°30 N; 0°50′–2°00′ E) (Figure 1). Originally designated a Game Reserve in 1954, it was subsequently upgraded to National Park status in 1961. Presently, the PBR encompasses a core area known as the “Pendjari National Park,” covering an expanse of 2660 km^2^, in addition to two adjoining hunting zones referred to as the “Konkombri” and “Pendjari” hunting zones, jointly covering 1971 km^2^. In the hunting zones, significant numbers of domestic livestock occur, with 618 animals recorded and a mean density of 31 individuals per herd, indicating substantial pastoral activity within the reserve (Antoninova et al. 2019). This livestock presence may lead to competition with wild grazers for forage, particularly during the dry season. The geographical boundaries of the PBR are defined by the Atacora mountain range to the east and the Pendjari River to the north and west. Within the PBR, six distinct habitat types can be identified, namely, Forest, Grassland, Rock vegetation, Shrub savanna, Tree savanna, and Woodland. The biodiversity within the Pendjari Biosphere Reserve is notably rich, featuring the presence of four of the “big five” iconic African species: the lion ( *Panthera leo* ), elephant ( *Loxodonta cyclotis* ), buffalo ( *Syncerus caffer brachyceros* ), and leopard ( *Panthera pardus* ). Additionally, the reserve hosts various herbivore species, including *Kobus kob, Redunca redunca, Kobus ellipsiprymnus defassa, Hippotragus equinus koba, Alcelaphus buselaphus major, Damaliscus lunatus korrigum, Tragelaphus scriptus, Sylvicarpa grimmia, Cephalophus rufilatus, Ourebia ourebi quadriscopa, Syncerus caffer brachyceros, Loxodonta cyclotis, Hippopotamus amphibius niger, and Phacocherus porcus* (Kassa et al. 2007). The density of some species in the Pendjari Biosphere Reserve is as follows: Baboon (1.15 ind./km^2^), Hartebeest (1.28 ind./km^2^), Buffalo (9.65 ind./km^2^), Common duiker (2.48 ind./km^2^), Kob (5.91 ind./km^2^), Bushbuck (0.47 ind./km^2^), Roan (2.06 ind./km^2^), Oribi (1.77 ind./km^2^), Warthog (3.59 ind./km^2^), and Bohor reedbuck (4.02 ind./km^2^) (Nago et al. 2016).

In the West African area, the fire season extends from October to May (Laris 2005; Zoffoun et al. 2023).

### 2.2 Data Collection

#### 2.2.1 Species Data Collection

The present study focuses on medium‐sized grazers (with more than 80% grass in their diet), including hartebeest (*Alcelaphus bucelaphus major*), kob ( *Kobus kob kob* ), korrigum or topi (*Damaliscus lunatus korrigum*), roan antelope ( *Hippotragus equinus* ), and waterbuck ( *Kobus ellipsiprymnus defassa* ) (Table 1). To conduct this research, we used occurrence and abundance data obtained from the total aerial mammals count performed by African Parks, the current manager of the Pendjari Biosphere Reserve. The survey was executed using transects that were 300 m wide on both sides of the aircraft. This specific distance was selected as it represents the maximum range at which the species under study could be reliably detected (Antoninova et al. 2019). Surveys were conducted using high‐wing Cessna aircraft flown at a constant altitude of 92 m (300 ft). Two trained rear‐seat observers independently monitored calibrated 300 m strips on each side of the aircraft, guided by wing‐strut markers indicating strip limits. Observers systematically scanned from the transect centreline outward and called out all detections, ensuring continuous visual coverage beneath the aircraft and minimizing the risk of missed animals (Antoninova et al. 2019). The count may result in an undercount of total animal numbers, as individuals directly beneath the Cessna along the transect centre may be missed, but this will not lead to bias in the measures of habitat use by the animals. The survey was conducted at the end of the dry season, spanning from late April to early May 2019. This timing was chosen because visibility is significantly improved due to reduced leafiness of trees and decreased airborne dust during this period. The flight paths (transect orientations) were positioned perpendicular to the main river flows and other ecological gradients. Browsers were not included in this study because their small body size made them difficult to detect from the aircraft, which could have compromised accurate species identification, thus only grazers were considered. We did not include elephants and hippopotamuses in this study because they are not strict grazers: elephants are mixed feeders with strong browsing behavior, particularly outside the wet season, while hippopotamuses exhibit nocturnal grazing and strong dependence on permanent water bodies. Buffalo and warthog were also excluded because their habitat selection is strongly influenced by factors other than fire regime, including social behavior, wallowing and rooting activities, and proximity to water, which could confound the specific role of fire in shaping grazing habitat selection.

**TABLE 1:** Species under study and their body mass.

Species	Scientific name	Body mass (kg)	UICN Red list status
kob	*Kobus kob kob*	85.8	Vulnerable (VU)
Korrigum	*Damaliscus lunatus korrigum*	127	Endangered (EN)
Hartebeest	*Alcelaphus bucelaphus major*	161	Vulnerable (VU)
Waterbuck	*Kobus ellipsiprymnus defassa*	215	Near Threatened (NT)
Roan	*Hippotragus equinus*	261.3	Least Concern (LC)

*Note:* Body mass data are taken from Kingdon (2015) and refer to the average weight of male and female. IUCN Red list status was accessed online at https://www.iucnredlist.org/ (last assessed 5 January 2026).

#### 2.2.2 Fire Regime Variable Collection

Fire was globally recognized to be a factor responsible for the abundance and distribution of herbivore species, especially in savanna ecosystems (Eby et al. 2014; Souza et al. 2023). For this study we used five fire‐related variables: fire frequency, fire age, burned < 1 year, burned < 2 years and burned > 10 years that have been demonstrated to be the important fire variables to predict habitat used by herbivore species (Souza et al. 2023) (Table 2). We extracted fire‐related variable values for each target species occurrence.

**TABLE 2:** Characteristics of variables used for grazer species abundance modelization in the Pendjari Biosphere Reserve (PBR).

Variables	Description	Variable type	Sources
Very recently burned (< 1 year)	Area that burned recently in less than 1 year	Binary factor: 1—yes, 0—no	MODIS MCD64A1 fire data
Recently burned (< 2 years)	Area that burned recently in less than 2 years	Binary factor: 1—yes, 0—no	MODIS MCD64A1 fire data
Burned (> 10 years)	Areas that have experienced a fire since more than 10 years	Binary factor: 1—yes, 0—no	MODIS MCD64A1 fire data
Fire frequency	Number of times the area burned in the last 20 years	Continuous variable, starting at 0	MODIS MCD64A1 fire data
Fire age	Number of year since when a fire occurred in the area	Continuous variable, starting at 0	MODIS MCD64A1 fire data
Location in the protected area	Occurrence in the Pendjari National Park or Hunting zone	2‐level factor: National Park, Hunting zone	PBR shapefile
NDVI (Normalized Difference Vegetation Index)	Vegetation wellness index	Continuous variable, comprise between −1 and 1. −1 ≤ NDVI ≤ 1	Landsat 8
Grazer's species abundance	Number of individuals per occurrence	Continuous variable, starting at 1	Aerial survey in PBR in dry season 2019

We download the MODIS monthly burned‐area product MCD64A1 v.1 freely available on https://earthexplorer.usgs.gov/ (last accessed October 12, 2022) to extract fire related variables. MODIS MCD64A1 fire data is a monthly, global gridded 500 m product containing per‐pixel burned‐area and quality information (Giglio et al. 2021). MODIS MCD64A1 detects the approximate dates of burning and maps the spatial extent of monthly fires. We therefore assess the fire related variable for each occurrence in the spatial distribution of 500 m enough to assess the fire related impact on herbivore species (Souza et al. 2023).

- Very recently burned (< 1 year) represent the area that burned recently in less than 1 year (in the current season of the study 2018–2019) and is a bimodal variable, 1 if the area is recently burned in the season and 0 if the area is burned for more than 1 year. We summarized the monthly burned area in the last 1 year from the study period using MODIS data, reclassify to obtain the burned < 1 year variable and then extract the value for each occurrence.
- Recently burned (< 2 years) designates areas that have experienced a fire within the two‐year period leading up to the study period in April 2019. This variable is binary, taking the value of 1 if the area was burned within the last 2 years and 0 if it was burnt more than 2 years ago. We compiled monthly burned area data from the past 2 years prior to the study period using MODIS data, reclassified it to derive the “Recently Burned (< 2 Years)” variable, and subsequently extracted the corresponding values for each species occurrence.
- Burned > 10 years variable designates areas that have experienced a fire since more than 10 years from the study period in April 2019. This variable is binary, taking the value of 1 if the area was not burnt within the last 10 years and 0 if it was burnt less than 2 years ago. We compiled monthly burned area data from the past 2 years prior to the study period using MODIS data, reclassified it to derive the “Burned (> 10 years)” variable, and subsequently extracted the corresponding values for each species occurrence.
- Fire frequency (number of times the area burned in the last 20 years) for each occurrence was extracted on MODIS MCD64A1 fire data available using the methodology described by Zoffoun et al. (2023). For each month, burned pixels were coded with a value of 1 and unburned pixels with 0. Monthly binary rasters covering the 20 years period were then summed pixel by pixel, so that the final raster represents fire frequency, with each pixel value indicating the total number of times that location experienced fire during the study period.
- The fire age variable is the number of years since a fire occurred in the area (0 when it occurred in less than 1 year) and obtained by extracting for each occurrence the last burned month and consequently determining the age to the study period time.

#### 2.2.3 Non Fire Related Variable Collection

The non‐fire related variable used in this study was the location in the protected area (Pendjari National Park (the core area) or in the Hunting Zone) as well as the NDVI (Normalized Difference Vegetation Index).

The location was extracted for each occurrence by projecting the occurrence on ArcGIS and the reserve layer and then extracting the location for each occurrence.

For NDVI, we calculated the index value using April 2019 Landsat 8 data (downloaded from https://earthexplorer.usgs.gov/ on March 15, 2022 at a spatial resolution of 30 m) using the formula below:

$$\text{NDVI}=\frac{\mathrm{NIR}-R}{\mathrm{NIR}+R}$$

NIR = Near Infra‐Red band and *R* = Red band.

NDVI is a composite proxy for total green vegetation, including the herbaceous layer (grasses and forbs) as well as the woody layer (shrubs and trees).

### 2.3 Data Analyses

In order to model grazer species' abundance in relation to fire regime variables and non‐fire related variables selected, we conducted a Generalized Linear Model (GLM) with Poisson distribution. The model was run for each focal species and helped to measure the relative importance of each habitat characteristic on the use by a particular species.

We defined the species abundance as the response variable in the model and the other variables as explicative variables (recently burned < 1 year, recently burned < 2 years, burned > 10 years, fire frequency, fire age, location and NDVI).

Habitat use by a particular species was modeled using the Poisson distribution as follows:

$$\log\left(\mu_{i}\right)=\beta 0+\beta 1X_{1}+\beta 2X_{2}+\ldots$$

where *μ*_*i*_ is a function of the explanatory variable ~ average value of the response for a group of *X* values, *β*0 is the constant (intercept), and *β*1 is the partial regression coefficient for variable *X*_1_, holding all other remaining predictors constant (Legler and Roback 2019). That is, e^β1^ represents the ratio of means for a one‐unit increase in *X*_1_.

We used the glm function from the lme4 package (Bates et al. 2015) to run the GLM in R 4.2.1 (R Core Team 2021). Poisson model assumes that the variance is equal to the mean. As it is stated overdispersion can occur when variance is much higher than mean (Legler and Roback 2019). We assess the Goodness‐of‐fit of the model by checking for overdispersion using the Pearson's chi‐squared test (Bolker et al. 2009). We therefore used the negative binomial distribution for all species model using glm.nb function from the MASS package, due to the overdispersion of Poisson distribution model (*p* < 0.05), and finally considered our data without overdispersion.

**FIGURE 2:**
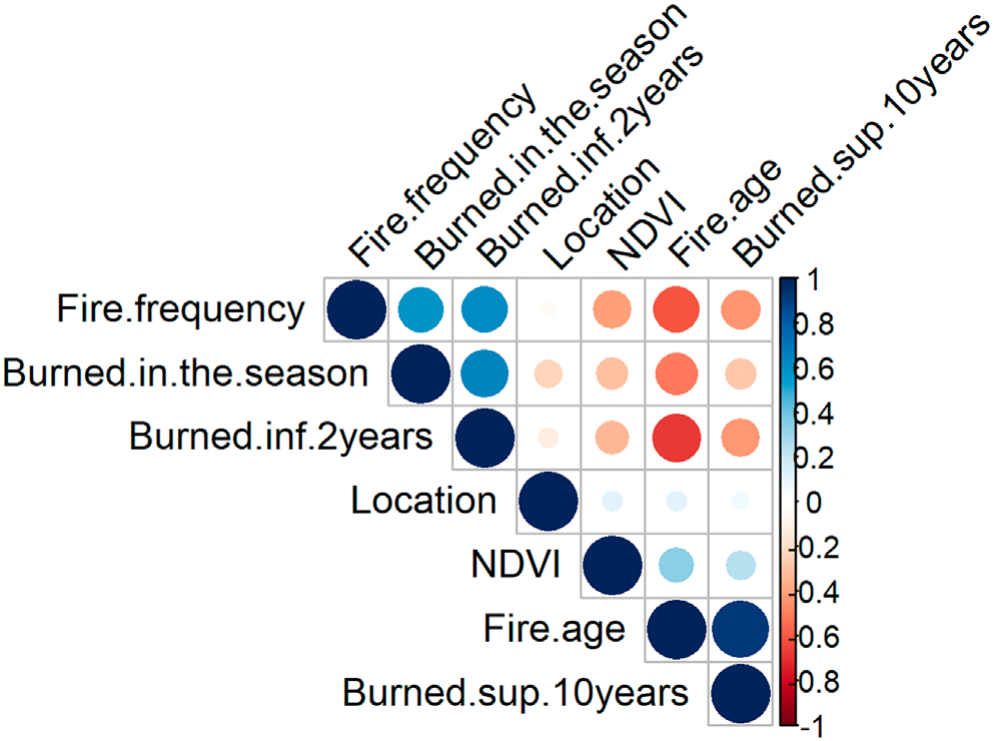
Correlation graphic between variables. The size of the circle indicates the level of correlation and the color the direction of the correlation, positive blue and negative orange. Burned in the season = burned < 1 year.

Collinearity among the predictor variables was investigated by examining the Pearson correlation coefficients between the measured variables (Figure 2). Correlations between NDVI and the other variables were low (*r* < 0.5). For fire‐related variables, correlations ranged from 0.40 to 0.67, except between fire age and fire frequency, which showed a very high correlation (*r* = 0.92, *p* < 0.05). As a result, we removed the variable burned > 10 years in the model to avoid the impact that collinearity could have on the models.

To identify the variables influencing species abundance, we used AICc to compare various models (Souza et al. 2023). Variables within models exhibiting ∆AICc < 2 were considered strongly supported for the species abundance prediction. When multiple models found support in the data, model averaging was employed to evaluate the beta estimates and their corresponding unconditional standard deviations. For model averaging, we exclusively considered models with substantial support (∆AICc < 2) following the criteria proposed by Burnham and Anderson (2002). Additionally, we calculated the relative importance of predictor variables by aggregating the weights of models in which the variable was a component, focusing on models with ∆AICc < 2.

All the aforementioned analyses were carried out using the statistical software R 4.2.1 (R Core Team 2021), with a significance threshold of 0.05 (alpha level).

## 3 Results

### 3.1 Occurrence of Grazers Species in Recently Burned Areas

**FIGURE 3:**
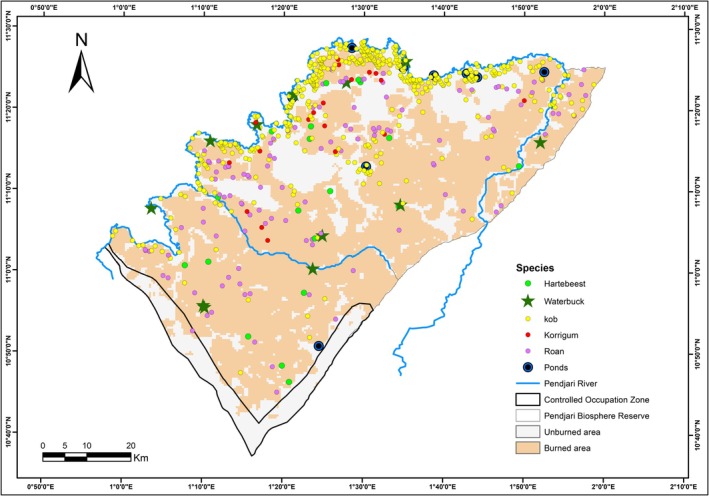
Species distribution in Pendjari Biosphere Reserve. Unburned area and burned area represent the area that didn't burn and burned respectively during the fire season 2018–2019 when the data was collected.

In the Pendjari Biosphere Reserve, there is a small variation in the percentage of occurrence of burned habitat among grazers species. While almost all species mainly used burned habitat in the fire seasons of data collection 2018–2019 (85.00%, 81.30%, 78.94% and 52.77% of occurrences respectively for korrigum, roan, hartebeest and Kob), less occurrence of waterbuck was found in the burned area (41.66%) (Figure 3, Appendix 1). However, among the 58.34% of waterbuck occurrences found in unburned area, 73.68% were located near a water source (< 500 m) (Figure 3).

### 3.2 Grazer Species Abundance and Space Use According to Fire Regime Variables in the PBR

Korrigum was only found in Pendjari National Park (PNP), while other studied grazer species (roan, kob, hartebeest and waterbuck) were found in both the national park (PNP) and Hunting zones (HZ). The location (PNP or HZ) in the reserve was a strong component in the model of kob and waterbuck (∆AICc < 2). As well, NDVI (a proxy for vegetation cover), a non‐fire regime variable, affected grazer species abundance (Table 3). The best models selection (∆AICc < 2) indicated an effect of the NDVI on the abundance of all grazer species studied. NDVI appears in three of the best models for kob, in two for roan and hartebeest, and in one for waterbuck and korrigum.

**TABLE 3:** Negative binomial model explaining grazer species habitat use (considered in term of abundance) in the Pendjari Biosphere Reserve according to the variables tested.

Models	k	AICc	∆AICc	AICc Wt	Cum.Wt	‐2LL
*Hartebeest*						
NDVI	2	117.23	0.00	0.26	0.26	−109.63
Fire age + Burned < 2 years	4	117.35	0.12	0.24	0.50	−106.49
Fire age	2	118.23	1.00	0.16	0.66	−110.63
Fire frequency	2	118.82	1.59	0.12	0.78	−111.22
Fire age + NDVI	3	118.83	1.60	0.12	0.89	−107.97
Burned < 2 years	3	118.96	1.73	0.11	1.00	−111.36
*Kob*						
Fire frequency + NDVI	3	2004.68	0.00	0.26	0.26	−1996.59
Burned < 1 year + Fire frequency + NDVI	5	2005.28	0.60	0.19	0.45	−1995.14
Fire frequency	2	2005.30	0.62	0.19	0.64	−1999.24
Fire frequency + Burned < 1 year	4	2005.78	1.10	0.15	0.79	−1997.69
Burned < 1 year + NDVI	4	2006.46	1.78	0.11	0.90	−1998.36
Location/PNP + Fire frequency + NDVI	5	2006.61	1.93	0.10	1.00	−1996.47
*Waterbuck*						
Location/PNP	3	68.59	0.00	0.31	0.31	−59.59
NDVI	2	69.88	1.29	0.16	0.47	−60.88
Burned < 2 years	3	70.23	1.64	0.14	0.61	−61.23
Fire age	2	70.30	1.71	0.13	0.74	−61.30
Burned < 1 year	3	70.36	1.77	0.13	0.87	−61.36
Fire frequency	2	70.37	1.78	0.13	1.00	−61.37
*Korrigum*						
Fire frequency	2	127.08	0.00	0.21	0.21	−119.58
Burned < 1 year	3	127.12	0.04	0.21	0.43	−119.62
NDVI	2	127.27	0.19	0.20	0.62	−119.77
Fire age	2	127.32	0.23	0.19	0.81	−119.82
Burned < 2 years	3	127.34	0.26	0.19	1.00	−119.84
*Roan*						
Fire age + Fire frequency + Burned < 2 years	5	711.64	0.00	0.24	0.24	−701.13
Burned < 1 year + Burned < 2 years + Fire frequency	6	712.13	0.49	0.19	0.43	−701.62
Burned < 1 year + Burned < 2 years	5	712.19	0.55	0.18	0.61	−703.85
Fire frequency + Burned < 2 years	4	712.27	0.63	0.17	0.78	−703.94
Fire age + Fire frequency + Burned < 2 years + NDVI	6	712.94	1.30	0.12	0.90	−700.22
Burned < 1 year + Burned < 2 years + Fire frequency + NDVI	7	713.48	1.83	0.10	1.00	−700.75

*Note:* Only models with ∆ AICc < 2 are shown. K represents the number of parameters in the model, AICc Wt and Cum. Wt indicate the relative weight of the model and the accumulated weight, respectively, and −2LL is the log‐likelihood.

Considering the spatial variation of fire regime variables, all grazers' species abundance was affected by fire related variables (Table 3). Models containing fire regime variables showed high support for all species abundance modelisation (∆AICc < 2). Furthermore, model selection showed that models containing fire related variables were ranked the top best model for kob, korrigum and roan, while the model containing fire related variables was the top 2 best model with ∆AICc < 0.5 for the hartebeest and the top 3 best model for waterbuck. This suggests that all grazer species considered use the habitat according to the spatial distribution of the fire regime. Fire frequency appears to be the variable that affected the most the abundance of all grazer species considered (Table 3) and was among the best models for all grazer species (∆AICc < 2).

**FIGURE 4:**
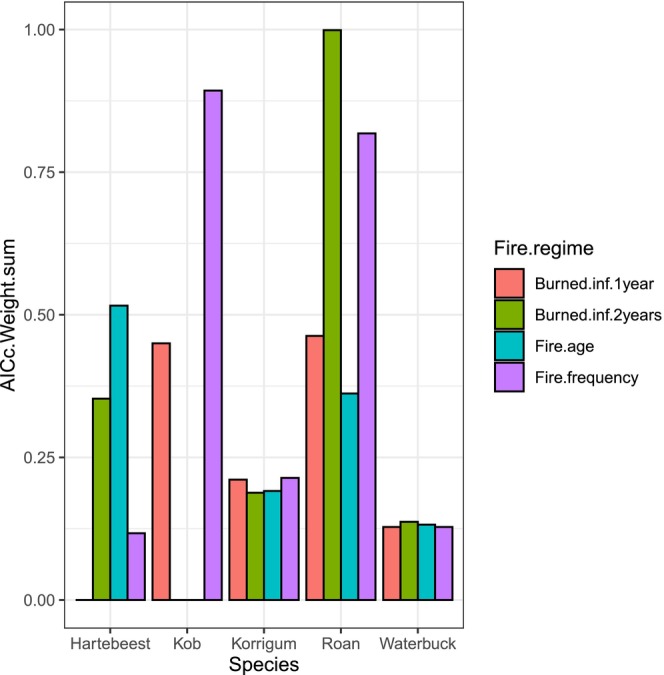
Relative importance of fire regime variables on abundance of grazer species (intensity of habitat use) of hartebeest—*Alcelaphus bucelaphus major*, kob—*Kobus* *kob kob* , korrigum—*Damaliscus lunatus korrigum*, roan—*Hippotragus* *equinus* and waterbuck—*Kobus* *ellipsiprymnus defassa* in the Pendjari Biosphere Reserve. Variable value of importance was calculated as the sum of model weights with ∆AICc < 2 that included the variable. Burned.inf.1 year: Burned < 1 year, Burned.inf.2 years: Burned < 2 years.

All the four fire regime variables considered for this study highly contributed (∆AICc < 2) to the grazer species abundance model of korrigum, waterbuck and roan, while only burned < 1 year and fire frequency contributed for kob model and burned < 2 years, fire age and fire frequency for hartebeest model (Figure 4, Table 3).

The relative importance of fire‐related variables in the use of habitat (considered in terms of abundance) by korrigum and waterbuck was low (weight sum < 0.25) for all variables (Figure 4). Fire frequency and fire age were the most frequent variables in the models with high support (∆AICc < 2). As a result, fire age was ranked the best fire‐related variable predictor for hartebeest abundance (weight sum > 0.50). And fire frequency was the best fire‐related predictor for the three over grazers species' abundance: kob (weight sum > 0.80), korrigum (weight sum < 0.25), and roan (weight sum > 0.80). Very recently burned (< 1 year) was rank second fire regime variable predictor for kob and third for roan but with weight sum > 0.45. Finally, recently burned (< 2 years) was rank best fire regime predictor for roan abundance (weight sum = 1) as well as waterbuck (weight sum < 0.25) (Figure 4).

**FIGURE 5:**
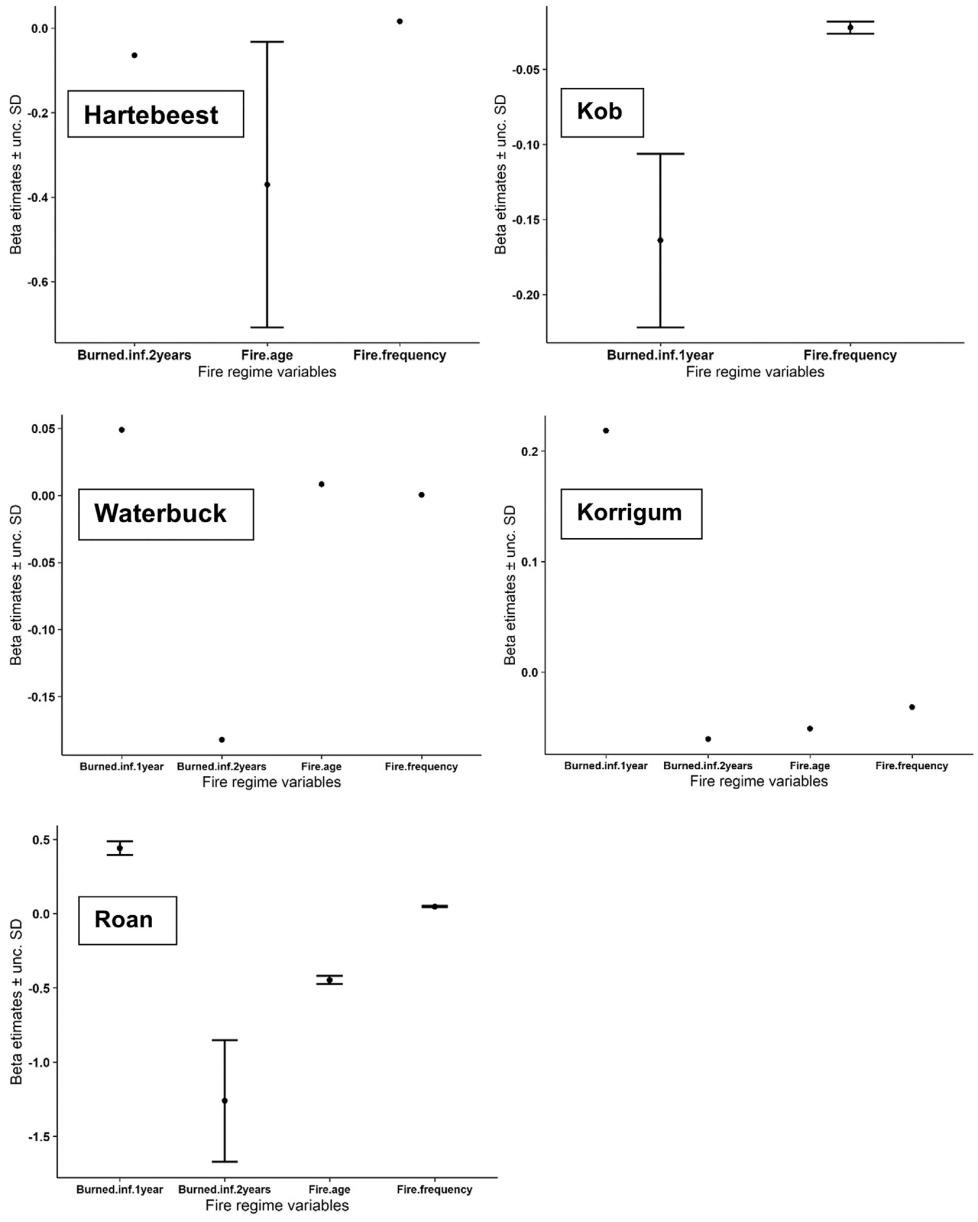
Mean coefficient estimates (β) and unconditional standard deviations (unc. SD) for the effect of fire regime variables on abundance of grazer species in the Pendjari Biosphere Reserve. β and unc. SD were based on model averaging of models with ΔAICc < 2.

By assessing the beta estimate value of each fire related variable highly affecting the abundance of grazer species (Figure 5), we found that very recently burned (< 1 year), fire age and fire frequency positively affected waterbuck abundance while recently burned (< 2 years) negatively affected its abundance. However, the mean beta estimates of fire age and fire frequency were negligible (≈0). That is, waterbuck species highly used space when the habitat is very recently burned (< 1 year). As for hartebeest, beta estimate indicates negative support to fire age and burned < 2 years, and positive support to fire frequency. Although, fire frequency and burned < 2 years have insignificant beta estimates (≈0). As a result, hartebeest species preferred recently burned space with low fire age. Kob responds negatively to fire frequency and burned < 1 year. Kob species showed a spatial distribution with high abundance very close to water sources and resulted in a preference for low frequently burned area; therefore, that did not account for the selection of fire relative variable.

Korrigum respond negatively to fire age, fire frequency, burned < 2 years and positively to burned < 1 year. However, fire age, fire frequency, burned < 2 years have insignificant beta estimates (≈0). In consequence, korrigum only really have been considerably positively affected by burned < 1 year. That means that the species abundance is high in areas that burned very recently (< 1 year). Lastly, roan species showed preference for very recently burned areas (< 1 year), with high fire frequency and thus low fire age; consequently, their abundances are high in those areas.

## 4 Discussion

Inference from habitat‐selection models is strongly dependent on sample size. In this study, occurrence data were sufficient for kob (435 records) and roan (123 records), allowing relatively robust interpretation of results for these species. In contrast, occurrence numbers were low for hartebeest (19 records), waterbuck (12 records), and korrigum (20 records), which limit statistical power and increase uncertainty in model selection and parameter estimates. Results for these species are therefore informative but based on limited data, and should be interpreted cautiously.

The present study showcases the fact that almost all grazer species studied (hartebeest, Kob, korrigum, and roan) have higher occurrences in burned habitats than in unburned habitats in the study season while waterbuck exhibited a different pattern, favoring areas in proximity to water sources which are mainly unburned. The observation that hartebeest, Kob, korrigum, and roan primarily selected burned habitats during the fire seasons aligns with the findings of previous research. Indeed, several studies have highlighted the positive impact of fire on grassland ecosystems, promoting the growth of fresh, nutrient‐rich vegetation that is favored by grazers (Archibald et al. 2005). These findings suggest that these species are likely benefiting from the increased forage quality and availability in recently burned areas (Nieman et al. 2021). Eby et al. (2014) showed that grazer species significantly selected burned habitat up to 60 days after fire. Murray and Brown (1993) showed that during the dry season in the Serengeti, topi (*Damaliscus lunatus jimela*, the local relative of korrigum) and hartebeest are highly selective for green grass leaves of high forage quality more than others grazers, which are typically found in recently burned areas. Recent studies confirm that grazers across African savannas respond strongly to recently burned patches, where forage quality is highest, but that this response is often time‐limited and influenced by grazing intensity and landscape heterogeneity (Karp et al. 2024; Masudi et al. 2024). Conversely, the lower occurrence of waterbuck in burned areas may be linked to their ecology. Waterbucks primarily inhabit regions abundant in water within the savanna ecosystem and favor woods or thickets, where they inhabit the fringes of valley grasslands, that usually doesn't burn (Kassa et al. 2007; Sinclair et al. 2000; Kingdon 2015). Thus, the observation that a significant proportion of waterbuck occurrences were in unburned areas near water sources in valley grasslands is consistent with their ecological requirements, and not really related to preference for unburned area.

Several abundance models per species have similar AICc values (ΔAICc < 2), except for the waterbuck. Habitat choice is not dominated by a single model for kob, roan, hartebeest, or korrigum. Instead, variance is distributed across multiple competing models, suggesting complex, multi‐factor decision‐making by grazers. Our results show that korrigum was exclusively found in Pendjari National Park (PNP), while other grazer species (roan, kob, hartebeest, waterbuck) were present in both PNP and Hunting Zones (HZ). Consequently, location does not appear as a predictor in korrigum models. This suggests that korrigum may be avoiding the Hunting Zones due to disturbance during the dry season. However, korrigum are now very rare in the reserve and are difficult to observe. The strong support for the models of kob and waterbuck in relation to designation location (PNP or HZ) with high abundance in PNP highlights the influence of habitat designation, thus hunting impact on their distribution. Given the near absence of poaching and hunting within the Pendjari National Park, this core area serves as a refuge where wildlife can find relative safety and tranquility. Such habitat specialization involving different ungulate species emphasizes the importance of core protected areas like PNP for conserving unique habitat niches. The core part of protected areas like PNP often provides enhanced habitat quality and reduced human disturbances, which can attract and support certain species (Davies et al. 2018), and hunting can have a detrimental effect on habitat selection by grazers, as found by Djagoun et al. (2014). Especially in the Hunting Zones of the PBR, the dry season when the data were collected corresponds to the period when hunting activities take place. Brooke et al. (2020) demonstrated that hunting disrupts ungulate responses to fire by limiting their ability to use optimal post‐fire forage, showing how anthropogenic pressures can confound fire–herbivore dynamics.

The significant effect of NDVI on the abundance of considered grazers underscores the importance of vegetation cover as a key driver of herbivore distribution (Pettorelli et al. 2014). NDVI serves as a proxy for vegetation moisture and availability, influencing forage selection by grazers. NDVI is relevant for grazers as it captures spatial variation in vegetation productivity, identifies greener patches likely to support higher forage availability, and is often correlated with recent regrowth, especially after fire or rainfall. The observed effect of NDVI on all species suggests that maintaining a healthy vegetation cover is essential for supporting diverse grazer populations in the reserve. This vegetation requirement for each species is therefore under constant influence by fire regime (Archibald et al. 2005).

The strong support for fire regime variables in the models of all grazer species highlights the role of fire in shaping habitat. The influence of fire regime variables on the abundance of grazer species within the Pendjari Biosphere Reserve is a crucial aspect of this study's findings. Fire can influence vegetation structure and composition, affecting the availability and quality of forage for herbivores (Smit et al. 2013). Fire is a key ecological driver in many savanna ecosystems, and its effects on herbivore populations have been widely documented in the literature. Numerous studies have highlighted the complex relationships between fire, vegetation, and herbivores. For example, a study by Archibald et al. (2005) in South Africa's Kruger National Park found that fire influenced the spatial distribution of herbivores by altering the quality and availability of forage. Fire frequency, in particular, emerged as a crucial variable affecting grazer abundance. Our findings are supported by Holdo et al. (2009), which demonstrated that fire frequency can strongly influence the abundance and distribution of herbivores in East African savannas, and Souza et al. (2023) who reported fire frequency as an important variable to predict the occupancy of herbivore species in the Neotropical Savanna of Brazil. Large‐scale analyses further highlight that herbivore use of burned areas depends not only on fire frequency but also on the interaction between fire age and grazing pressure, creating shifting mosaics of habitat use (Karp et al. 2024).

The assessment of the relative importance of fire‐related variables in shaping the space use and abundance of korrigum, waterbuck, hartebeest, kob, and roan provides valuable insights into the nuanced responses of these species to different aspects of the fire regime within the Pendjari Biosphere Reserve. For korrigum and waterbuck, the study found that fire‐related variables had a relatively low importance (weight sum < 0.25) in explaining their abundance. However, it's important to note that fire frequency and fire age consistently appeared in models with high support of korrigum and waterbuck (∆AICc < 2). While these variables may not be the primary drivers of abundance for korrigum and waterbuck, their presence in models with high support suggests that fire does play a role in shaping the spatial distribution and abundance of these species to some extent. In contrast, fire‐related variables emerged as more influential for hartebeest, kob, and roan. Notably, fire age was identified as the best predictor for hartebeest abundance, with a weight sum exceeding 0.50. This finding suggests that hartebeest populations respond significantly to the temporal aspect of fire, particularly the time since the last fire. This aligns with research by Tamrat et al. (2020), which demonstrated that hartebeest in East African savannas exhibit strong preferences for recently burned areas due to increased forage quality. Masudi et al. (2024) confirmed that post‐fire herbivory is highly dynamic, with herbivore movements tracking regrowth stages across different times since fire. For kob and roan, fire frequency stood out as the top‐ranked and second top‐ranked fire‐related predictor respectively, with weight sums exceeding 0.80. This indicates that these species are highly responsive to the frequency of fires within their habitat. Fire frequency is a critical factor affecting vegetation dynamics in savanna ecosystems, which, in turn, can influence herbivore abundance and distribution (Archibald et al. 2005). Very recently burned (< 1 year) was rank second fire regime variable predictor for kob and third for roan but with weight sum > 0.45 showing the influence of the variable on abundance of grazers species. Eby et al. (2014) showed that very recently burned area have a significant impact on grazer abundance. Finally, recently burned (< 2 years) was rank best fire regime predictor for roan abundance (weight sum = 1) as well as waterbuck (weight sum < 0.25). It indicates a contribution of prolonged impact of fire after the fire season.

The examination of beta estimate values for fire‐related variables sheds light on the specific preferences and responses of grazer species abundance to different aspects of the fire regime within the Pendjari Biosphere Reserve. Waterbuck, for instance, displayed a clear preference testified in their abundance in very recently burned areas (< 1 year). This aligns with previous research by Mahakata and Mapaure (2022), which observed that waterbuck tend to favor areas with fresh regrowth following fires, likely due to the increased availability of nutrient‐rich forage. Hartebeest, on the other hand, exhibited a preference for recently burned areas with low fire age. This preference for areas with younger fires is consistent with the findings of Tamrat et al. (2020), who noted that hartebeest often select grassland habitats with shorter fire return intervals, as these areas tend to have higher forage quality and palatability. Korrigum demonstrated a considerable positive response to areas burned within the fire season, indicating a preference for very recently burned habitats. This preference for freshly burned areas may be linked to the rapid post‐fire vegetation regrowth, which can provide abundant and nutritious forage for this species (Belsky 1994). Roan showed a complex response to fire‐related variables. While roan exhibited a negative response to areas burned within the past 2 years and older fires, it displayed a positive response to higher fire frequencies and areas burned within the fire season. This suggests that roan thrive in areas with a high frequency of recent fires, likely due to the increased availability of fresh forage. This aligns with the findings of Archibald et al. (2005), which highlight the positive impact of frequent fires on grazer abundance in savanna ecosystems. Moreover, Eby et al. (2014) found that overall, there is a significant difference between abundance of grazer species in very recently burned area and unburned area after fire and mainly the first 60 days after the fire. Comparative studies add that these species‐specific responses to fire mosaics create shifting patterns of occupancy at landscape scales, reinforcing the importance of managing for pyrodiversity (Souza et al. 2023).

The modification of the physical environment is one of the indirect effects of fire. Fire modifies the structure and composition of vegetation, which might affect resource availability, quality, and quantity for herbivores, particularly grazing herbivores (Mills and Fey 2005). Despite forage quantity being low, the available forage has improved forage protein, palatability, and digestibility, leading to a preference for burnt areas (Gureja and Owen‐Smith 2002; Fuhlendorf and Engle 2004). Another indirect impact of fire is that herbivores might prefer burned open areas because they are less vulnerable to predation due to greater visibility (Owen‐Smith 2008). Lastly, kob, in contrast, responded negatively to fire frequency and recently burned areas (< 1 year). The preference for less frequently burned areas may be more related to the ecology of the species, showing presence of habitats near water sources, which can mitigate the impact of fires. This observation aligns with the research of Antwi et al. (2018), which found that kob tend to aggregate around water sources in savanna ecosystems, especially in the dry season, as riparian areas provide both essential drinking water and higher‐quality forage. The beta estimate values provide valuable insights into how different grazer species within the Pendjari Biosphere Reserve respond to the different components of the fire regime. These preferences and responses are likely driven by the varying forage quality and availability associated with different fire histories and frequencies.

Our findings clearly indicate that fire frequency is the most influential fire variable shaping grazer abundance. Most grazers showed strong associations with very recently burned areas (< 1 year) and with landscapes experiencing relatively frequent fires, which likely maintain a continuous supply of short, high‐quality grass regrowth during the dry season. A fire management strategy that benefits grazers would therefore aim to maintain regular dry‐season fires to support forage quality associated with green grass leaves; ensure a mosaic of fire ages, including very recent burns and intermediate recovery stages, while maintaining lower fire frequency near permanent water sources; and use fire strategically to complement conservation objectives across different management zones (PNP vs. HZ).

## 5 Conclusion

Fire regime is an important driver of habitat selection by grazer species in west African savannas. All the grazer species studied abundance was intrinsically related to the frequent burning of the Pendjari Biosphere Reserve, showing the importance of fire to maintain this reserve's diversity. This research reveals the fire metric importance for each species‐specific grazer consideration. While some grazer species may exhibit a strong preference for recently burned areas, others may respond primarily to fire frequency or the time since the last fire. These insights are valuable for understanding the complex interactions between grazers and fire in savanna ecosystems. The results of this study emphasize the importance of considering fire regime variables when assessing the abundance and distribution of grazer species in savanna ecosystems. These findings align with broader ecological research on the role of fire in shaping herbivore communities and highlight the need for integrated fire management strategies in conservation efforts.

## Author Contributions

**Omobayo Ghislain Zoffoun:** conceptualization (lead), data curation (equal), formal analysis (lead), funding acquisition (equal), methodology (lead), supervision (equal), validation (lead), visualization (lead), writing – original draft (lead), writing – review and editing (equal). **Chabi A. M. S. Djagoun:** conceptualization (equal), validation (equal), writing – review and editing (equal). **Côme Agossa Linsoussi:** visualization (equal), writing – review and editing (equal). **Etotépé A. Sogbohossou:** supervision (equal), writing – original draft (equal), writing – review and editing (equal).

## Funding

This work was supported by the American Society of Mammologists (ASM).

## Ethics Statement

This study did not involve direct interaction with animals or manipulation of wildlife. All analyses were conducted using existing total aerial count datasets and publicly available satellite‐derived fire data. As such, formal animal ethics approval was not required. Data collection for the aerial surveys was conducted by the responsible authorities in accordance with national wildlife monitoring regulations and ethical standards.

## Conflicts of Interest

The authors declare no conflicts of interest.

## Data Availability

The original contributions presented in this study are included in the article; further inquiries can be directed to the corresponding author.

## Appendix 1 Number of Occurrences for Each Species in Burned and Unburned Habitat During the Fire Season 2018–2019 When the Data Was Collected

**Table:**

Species	Occurrences		Total occurrences
	Burned area	Unburned area	
Hartebeest	15	4	19
Kob	219	216	435
Waterbuck	5	7	12
Korrigum	17	3	20
Roan	100	23	123
Total	356	253	609
